# Detecting gene signature activation in breast cancer in an absolute, single-patient manner

**DOI:** 10.1186/s13058-017-0824-7

**Published:** 2017-03-21

**Authors:** E. R. Paquet, R. Lesurf, A. Tofigh, V. Dumeaux, M. T. Hallett

**Affiliations:** 1 Centre for Bioinformatics, McGill University, Montreal, Quebec H3G 0B1 Canada; 2 The Rosalind and Morris Goodman Cancer Research Centre, McGill University, Montreal, Quebec H3A 1A3 Canada; 3 School of Computer Science, McGill University, Montreal, Quebec H3A 0E9 Canada

**Keywords:** Breast cancer, Single sample, RNA-seq, Gene expression, N-of-1, Absolute assignments and pathway activation

## Abstract

**Background:**

The ability to reliably identify the state (activated, repressed, or latent) of any molecular process in the tumor of a patient from an individual whole-genome gene expression profile obtained from microarray or RNA sequencing (RNA-seq) promises important clinical utility. Unfortunately, all previous bioinformatics tools are only applicable in large and diverse panels of patients, or are limited to a single specific pathway/process (e.g. proliferation).

**Methods:**

Using a panel of 4510 whole-genome gene expression profiles from 10 different studies we built and selected models predicting the activation status of a compendium of 1733 different biological processes. Using a second independent validation dataset of 742 patients we validated the final list of 1773 models to be included in a *de novo* tool entitled absolute inference of patient signatures (AIPS). We also evaluated the prognostic significance of the 1773 individual models to predict outcome in all and in specific breast cancer subtypes.

**Results:**

We described the development of the *de novo* tool entitled AIPS that can identify the activation status of a panel of 1733 different biological processes from an individual breast cancer microarray or RNA-seq profile without recourse to a broad cohort of patients. We demonstrated that AIPS is stable compared to previous tools, as the inferred pathway state is not affected by the composition of a dataset. We also showed that pathway states inferred by AIPS are in agreement with previous tools but use far fewer genes. We determined that several AIPS-defined pathways are prognostic across and within molecularly and clinically define subtypes (two-sided log-rank test false discovery rate (FDR) <5%). Interestingly, 74.5% (1291/1733) of the models are able to distinguish patients with luminal A cancer from those with luminal B cancer (Fisher’s exact test FDR <5%).

**Conclusion:**

AIPS represents the first tool that would allow an individual breast cancer patient to obtain a thorough knowledge of the molecular processes active in their tumor from only one individual gene expression (N-of-1) profile.

**Electronic supplementary material:**

The online version of this article (doi:10.1186/s13058-017-0824-7) contains supplementary material, which is available to authorized users.

## Background

There are many pathway analysis approaches that seek to determine whether a specific molecular process or cellular response is activated, repressed, or latent in a given patient sample including pathway level analysis of gene expression (PLAGE) [1], zscore [2], single sample gene set enrichment analysis (ssGSEA) [3], functional analysis of individual microarray expression (FAIME) [4], gene set variation analysis (GSVA) [5], Pathifier [6], N-of-1 [7] and diversity arrays technology (DART) [8]. To make predictions, all of these methods require a suitable database of gene signatures, where each signature is composed of a set of genes with expression levels that correlate with different activation states of the specific molecular process or biological response. Typically, these approaches return a score that measures the level of activation of the process or response.

There are important limitations associated with these existing approaches. First, the vast majority (ssGSEA, GSVA, Pathifier, FAIME and DART) generate scores that are not easily interpreted in isolation. More specifically, the score for an individual pathway in a tumor must be interpreted relative to the distribution of scores for all patients in a large cohort. This is certainly problematic for situations where only the sample from the target patient is available without recourse to a large dataset of patients for interpretation, a scenario that often occurs in clinical investigations and in the clinic.

Second, the relativistic nature of some of the existing methods makes them highly sensitive to the specific composition of the patient’s dataset [9, 10]. For example, scores for a given pathway in a specific patient will vary depending on the clinicopathological and molecular characteristics of the other tumors/patients in the dataset. This is analogous to the instability that was previously described for methods that determine breast cancer subtype when the fraction of patients with estrogen receptor (*ER*)-positive or *HER2*-positive cancer varies between datasets [9, 10]. Techniques such as N-of-1 [7] are potentially not as prone to these problems. However, they require both a normal and a tumor sample from the same patient, which is difficult to obtain and is not yet standard clinical practice.

Ideally, a tool to measure the activation of a pathway via a gene signature should have the following four essential properties: (1) it should be applicable to gene expression profiles of single patients without recourse to or need for a larger cohort for comparative analysis; (2) the calls should be “stable”, i.e. predictions should not be influenced by the composition of a comparative patient cohort; (3) it should function across a large range of platforms (e.g. various microarrays and RNA-seq technologies); and (4) the prediction of the state of each gene signature in a patient’s tumor should be justified statistically. Unfortunately, to our knowledge, there is no approach currently available that meets all these four criteria.

Here we present an approach entitled absolute inference of patient signatures (AIPS) that builds upon our previously described absolute intrinsic molecular subtyping (AIMS) method to predict the breast cancer subtype in a manner respecting the aforementioned properties 1–4 [9]. The absolute, stable and general properties of AIMS are generalized from markers of subtype classification to a large set of gene expression signatures representing an extremely diverse array of biological processes, pathways and functions, encompassing the hallmarks of breast and other cancers. The tool allows the molecular dissection of a single tumor for all relevant biological processes that are activated or repressed at the transcriptional level, and greatly extend the current technologies that measure just a single process (e.g. proliferation, immune-related information, etc.).

## Methods

Supplemental methods are provided in Additional file 1.

### Testing the stability of current approaches for assigning pathway activation scores

**Fig. 1:**
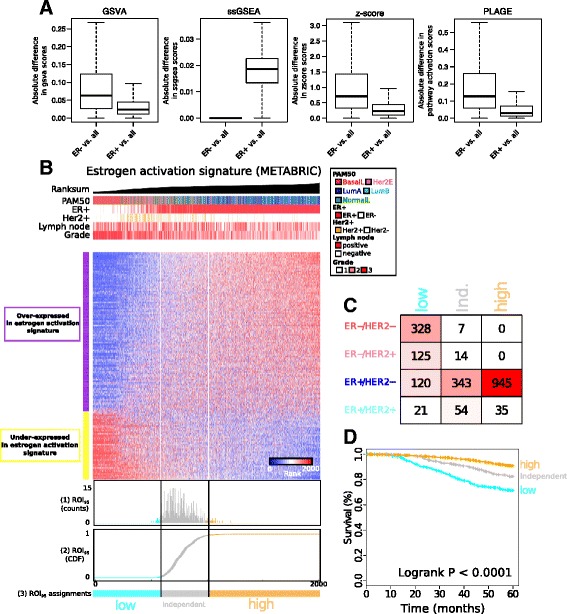
Instability of current pathway activation inference tools and example application of the region of independence (*ROI*). **a** Absolute difference between the score obtained from a specific pathway activation tool (gene set variation analysis (*GSVA*), single sample gene set enrichment analysis (*ssGSEA*), zscore, or pathway level analysis of gene expression (*PLAGE*)) using all patients from the Molecular Taxonomy of Breast Cancer International Consortium (METABRIC) dataset, and the score obtained when this dataset is restricted to either estrogen receptor (*ER*)-negative (*ER-*) samples (*left*) or *ER*-positive (*ER+*) samples (*right*). **b** Heatmap depicts the ROI induced over the rank-sum-base ordering of patients in the METABRIC dataset using the estrogen activation gene signature from Doane et al. with the probability (1) and cumulative distribution function (2) for the random trials, in addition to the final assignments (3) into low, high and independent regions (defined as the 95% CI of the index of the random trials). **c** Distribution of the low, independent and high assignments defined by the ROI_95_ in the function of the clinical subtypes (defined by ER and human epidermal growth factor receptor 2 (*HER2*) status). **d** Class assignments defined by the ROI_95_ are prognostic with 5 years survival (log-rank test *P* < 0.00001)

This analysis took advantage of the implementations of ssGSEA, PLAGE, zscore and GSVA in the GSVA Bioconductor package [5]. We restricted our attention to the 4725 gene signature in C2 (curated gene sets) from the MSigDB [11]. Using the complete Molecular Taxonomy of Breast Cancer International Consortium (METABRIC) gene expression dataset (*n* = 1992), we assigned gene signature activation scores using one of the four approaches for all gene sets in the C2 collections to all patients in METABRIC. This produces a matrix M_ALL_ in which M_ALL_ (P_i_,GS_j_) corresponds to the score assigned to patient P_i_ using gene set GS_j_. We repeated the score assignment a second time by applying the approach to only the ER-positive patients in the METABRIC dataset and obtained a second matrix of scores M_*ER*+_ in which M_*ER*+_(P_i_,GS_j_) corresponds to the score assigned to patient P_i_ using gene set GS_j_. We repeated this analysis for the ER-negative patients to obtain a third matrix M_ER-_. The distributions presented in Fig. 1a are obtained from the absolute difference between the M_ALL_ and M_*ER*+_ or M_*ER*-_ matrix. For example, the distribution for the *ER*-positive patients are computed by |M_ALL_(P_i_,GS_j_) - M_*ER*+_(P_i_,GS_j_)| for all *ER*-positive patients and all the gene sets GS_j_.

### Preparation of gene expression datasets

We utilized the same training set as described in Paquet et al. [9] with the exception of the dataset of Loi et al., because after careful examination, this dataset was mainly composed of *ER*-positive samples and had evidence of batch effects with *ER*-negative samples (Table 1). Briefly, we generated the training set by downloading all of the original normalized dataset from the Gene Expression Omnibus (GEO) and ArrayExpress (Table 1). The normalized data are used directly for the region of independence (ROI)_95_ assignments (see Additional file 1 for a description of the ROI_95_), but as AIPS requires quantification of raw gene expression, we also downloaded the raw gene expression data and pre-processed them to remove background artifacts when applicable (mostly for the microarray platforms).

**Table 1:** Characteristics of the breast cancer datasets used in this study

Dataset	Training/validation	Platform	Number of samples	*ER*+	*Her2*+	BasalL	*Her2* *E*	LumA	LumB	NormaL
expO Bittner M. (www.intgen.org)	Training	Affymetrix (U133 Plus 2.0)	312	65.7%	28.1%	21.2%	16.3%	31.4%	18.9%	12.2%
Lu et al. Breast Cancer Res Treat 2008 [40]	Training	Affymetrix (U133 Plus 2.0)	127	58.3%	23.6%	26.8%	17.3%	37.0%	16.5%	2.4%
Li et al. Nat Med 2010 [41]	Training	Affymetrix (U133 Plus 2.0)	115	60.9%	31.3%	27.0%	16.5%	36.5%	18.3%	1.7%
Parker et al. J Clin Oncol 2009 [25]	Training	Agilent	226	58.2%	12.4%	31.0%	12.4%	33.2%	16.4%	7.1%
Curtis et al. Nature 2012 (METABRIC) [13]	Training	Illumina (HT-12 v3)	1992	76.2%	12.5%	20.5%	16.0%	26.7%	22.8%	14.0%
Guedj et al. Oncogene 2012 [42]	Training	Affymetrix (U133 Plus 2.0)	537	75.9%	13.0%	16.2%	17.1%	24.8%	24.2%	17.7%
TCGA Nature 2012 [23]	Training	Agilent	233	79.3%	21.9%	22.3%	15.5%	30.9%	21.0%	10.3%
Miller et al. PNAS 2005 [43]	Training	Affymetrix (U133AB)	251	86.2%	13.1%	15.9%	18.3%	25.1%	20.3%	20.3%
Pawitan et al. Breast Cancer Res 2005 [44]	Training	Affymetrix (U133AB)	159	N/A	13.8%	12.6%	13.8%	28.3%	27.7%	17.6%
TCGA Nature 2012 [23]	Training	RNA-seq (Illumina)	558	77.9%	24.2%	19.2%	12.9%	30.5%	22.2%	15.2%
McGill [12]	Validation	Affymetrix Gene ST	429	78.1%	18.5%	20.5%	17.4%	37.6%	16.2%	8.1%
TCGA Nature 2012 [23]	Validation	RNA-seq (Illumina)	313	72.6%	11.9%	19.2%	13.4%	35.8%	15.3%	16.3%

*ER+* estrogen-receptor-positive, *Her2+* human epidermal growth factor receptor 2-positive, *BasalL* basal-like intrinsic subtype, *Her2E* Her2-enriched intrinsic subtype, *LumA* luminal A intrinsic subtype, *LumB* luminal B intrinsic subtype, *NormaL* normal-like intrinsic subtype, *RNA*-*seq* RNA sequencing, *METABRIC* Molecular Taxonomy of Breast Cancer International Consortium, *TCGA* The Cancer Genome Atlas

The McGill validation dataset was generated on the Human Affymetrix Gene ST platform as previously described in Tofigh et al. [12]. For the METABRIC dataset we kept the 12 replicate samples described by the authors of the original publication [13]. As these correspond to less than 0.2% of our training set, their inclusion does not significantly affect any of the presented results. The final analyses and models were restricted to the Entrez IDs present on all the platforms in the training and validation datasets. When multiple probes map to the same Entrez ID, the ROI_95_ assignments used the most variable probe (using the interquartile range (IQR)). Unfortunately, the AIPS models cannot rely on such an approach because the assignments must be performed in the context of only a single sample. Therefore, the IQR cannot be used. The AIPS models used the probe with the highest raw gene expression in downstream analyses. We favored this solution over taking the mean of all probes because we believe that using the mean would bias the approach by favoring genes with more probes, as they would have less variance. On the other hand, it could also introduce noise because we cannot really expect all the isoforms of a gene to have similar levels of expression.

### Assembling a large collection of harmonized gene signatures

**Fig. 2:**
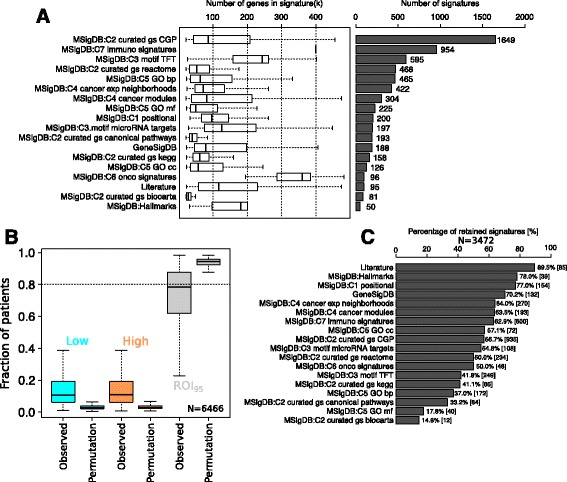
Selection of the informative list of 3472 gene signatures. **a** Distribution of the number of genes per signature and overall number of signatures from each sources of signatures. **b** Distribution of the proportion of patients assigned to the low, independent and high classes by the region of independence (ROI)_95_ computed for all signatures and obtained after random permutation of gene labels over the Molecular Taxonomy of Breast Cancer International Consortium (METABRIC) dataset. **c** Percentage of informative signatures for each of the different sources

We collected 6466 gene signatures from different sources including MSigDB (C1 to C7, *n* = 6183 [11]), GeneSigDB (*n* = 188, [14]), and breast-cancer-specific gene signatures obtained from the literature (*n* = 95). The number of signatures from each source is depicted in Fig. 2a. Entrez IDs were used in order to guarantee distinct, unambiguous gene names, identifications and symbols. As best possible, we determined the “directionality” of expression for each gene within each signature, and used this information to partition the genes into the overexpressed and underexpressed subsets.

### Development of AIPS

**Fig. 3:**
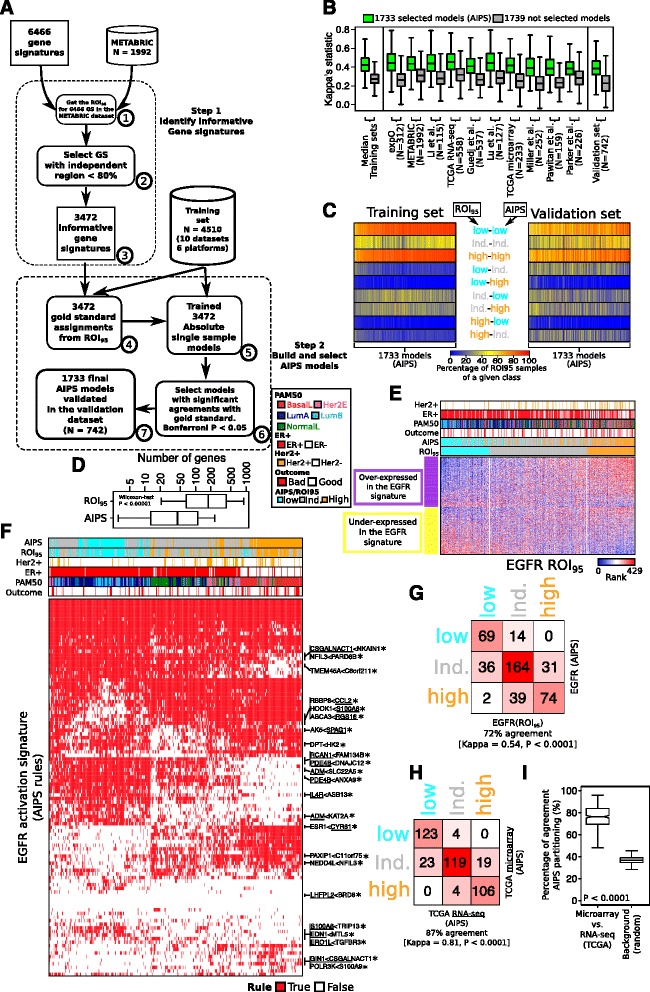
Generation and validation of the absolute inference of patient signatures (AIPS) models. **a** Pipeline used for the development of *AIPS*: (1) using our curated list of 6466 gene signatures, we used the region of independence (*ROI*)_95_ to obtain assignments in the Molecular Taxonomy of Breast Cancer International Consortium (*METABRIC*) dataset; (2) using a cutoff of 80% for the percentage of independent patients we selected the informative gene signatures (*GS*); (3) we obtained a list of 3472 informative gene signatures; (4) using the 4510 samples in our training set and the 3472 informative gene signatures we obtained the gold standard assignments for the training set; (5) using an approach similar to absolute intrinsic molecular subtyping (AIMS) (Paquet et al.) we trained 3472 absolute models mimicking the 3472 informative gene signatures; (6) we selected the final list of models that constitute AIPS by requiring significant agreement with the ROI_95_ assignments in all the individual datasets present in the training set; and (7) we validated the final list of 1733 AIPS models in the validation set. **b** Distributions of the kappa statistics for the selected 1733 models forming AIPS (*green*) and the 1739 models not forming AIPS (*gray*) in the entire training set (using the median of the individual training sets), the individual training sets, and the validation set. **c** Heatmaps depict the percentage of samples of a given class obtained from the ROI_95_ (e.g. low, independent (*ind.*), or high) assigned to another class by AIPS in the training and validation sets. **d** Number of genes utilized in the ROI_95_ versus the AIPS models. **e** The ROI_95_ example for an epidermal growth factor receptor (*EGFR*) signature from MSigDB in the McGill validation dataset. AIPS assignments are presented at the *top* of the heatmap. **f** Heatmap ordered using the Euclidean distance and the Ward’s linkage method presenting the different rules utilized in the AIPS-*EGFR* models (*red* means the rule is true and *white* means the rule is false). *Underlined* genes in rules marked by a *star* are enriched in genes upregulated by *EGFR* in MCF7 cell lines [22]. **g** Confusion matrix representing the agreement between the single sample AIPS-*EGFR* model and the whole-cohort ROI_95_ assignments. **h** Confusion matrix representing the agreement between the AIPS-*EGFR* assignments performed on the same RNA extraction but different platforms (RNA sequencing (*RNA-seq*) versus microarray). **i** Boxplots depicting the distribution of the percentage of agreement for the AIPS partitions done on The Cancer Genome Atlas (*TCGA*) samples profiled on both microarray and RNA-seq. We also present a background distribution generated from shuffling the labels 100 times. *ER* estrogen receptor, *HER2* human epidermal growth factor receptor 2, *PAM50* prediction analysis of microarray 50

The pipeline to generate AIPS models is summarized in Fig. 3a and contains two major steps: winnowing to informative gene signatures in breast cancer, followed by the selection of reliable absolute models.

### Winnowing to informative gene signatures in breast cancer

The numbers refer to Fig. 3a.

1. We applied the ROI_95_ on the 6466 gene signatures in our collection using the largest of our gene expression datasets (METABRIC, *n* = 1992). The size of the dataset helps to ensure that the ROI_95_ will have sufficient samples to precisely identify the region of independence (see Additional file 1 for a description of the ROI_95_)
2. To estimate significance, we also performed the same analysis but first randomly permuted the genes in the METABRIC dataset prior to applying the ROI_95_. In this manner, we are able to identify those gene signatures that behave essentially like random gene signatures. For every gene signature in the non-permuted versus permuted dataset we generated distributions from the percentage of patients assigned to the low, independent or high classes (Fig. 3b)
3. We selected a value of 0.8 to apply to the percentage of patients in the independent class as a cutoff to filter out non-informative gene signatures. This conservative cutoff still induces a sufficient number of patients within each of the three classes (low, independent, and high) to enable training of our models. A 0.8 cutoff on the percentage of independent assigned patients for a given signature retains 3472 gene signatures, which we consider to be informative in breast cancer (Fig. 2c).

### Building and selecting reliable absolute models from the informative gene signatures

(4) Our gene expression training set consists of 4510 patients across different datasets generated via six different technological platforms (Table 1). The ROI_95_ was applied to each signature on each individual normalized dataset in the training set. Variability in the patient composition across the different datasets (e.g. heterogeneity in receptor status or other clinical variables; Table 1) and the diverse range of platforms in our training set rules out an approach that builds a merged normalized meta-dataset
(5) Step (4) provides statuses (low, independent, and high) for 3472 signatures in each of 4510 patients. A model is learnt for each such signature as follows. First, we consider every possible pair of genes *x* and *y* in the expression dataset, and we ask how strongly the rule “if expression of *x* is greater than *y*, then classify signature as high” is associated with the ROI_95_ assignments. This is repeated for a modification of the rule where *x* is less than *y*, and for the other activation statuses of independent and low. This process is performed on the entire training set and weights are assigned to every patient in a way to give equal weight to the individual datasets i.e. patients from smaller datasets are given more weight than patients from larger datasets. Then, for each signature, the *K* most highly associated rules are chosen from the ranked list of all possible gene pair rules. The optimal number of rules *K* is chosen using 10-fold cross-validation for each model. The final *K* rules are combined into a single probabilistic model via a naive Bayes’ classifier
(6) Following the generation of models corresponding to absolute versions that mimic the ROI_95_ in step (5), we remove those models that do not achieve sufficiently high agreement with the gold standard ROI_95_ (Bonferroni-adjusted *P* value <0.05 on kappa statistics, Fig. 3b). Given the fact that many or most of the gene signatures in our database were constructed in contexts outside of breast cancer or cancer in general, there is no reason to expect all gene signatures to be informative and to induce good absolute models. After this filtering, we have 1733 absolute models that well mimic the ROI_95_ gold standard in the training set. This set constitutes the final version of AIPS.
(7) The final list of 1733 models (AIPS) was applied to the validation set and their agreement compared to the agreement in the training set (Fig. 3b).

### Kallisto and RSEM on single RNA-seq profiles

We used Kallisto version 0.42.4, RSEM version 1.2.15 and bowtie2 version 2.1.0. We ran Kallisto on the ensembl transcripts build provided on the Kallisto website (Homo_sapiens.GRCh38.rel79.cdna.all.fa) using the command “kallisto quant -i Homo_sapiens.GRCh38.rel79.cdna.all.idx --plaintext”. We ran RSEM on the UCSC hg19 gene annotation (2014-06-02) using rsem-calculate-expression in paired-end mode.

### Statistical analyses

All the statistical tests performed in this study are two-sided. For the log-rank test and survival analyses we used the package “survival” in R version 2.15. For analysis of Cohen’s kappa statistics and the significance we used the implementation available in the “fmsb” R package. All the visualizations were performed using custom R scripts.

We ran the survival analyses for the 1733 AIPS models on different cohorts of patients defined using either the molecularly defined subtype or the clinically defined subtypes. We also ran association analyses to study the association between the clinical and molecular subtypes and the low, independent, and high partitioning obtained from AIPS. For the proliferation score we used the Kruskal–Wallis test to evaluate the association with AIPS partitioning. We combined the *P* values obtained from both the survival analyses and and analyses of association using the Benjamini-Hochberg method to obtain a final false discovery rate (FDR). We considered an event to be significant with FDR <5%.

## Results

### Current single sample pathway assignment tools are unstable

The currently available tools including PLAGE [1], z-score [2], ssGSEA [3], and GSVA [5] infer activation scores for a given patient relative to a large cohort of patient profiles. We asked if, and to what degree, scores determined in this relativistic manner, were affected by perturbations to the composition of patients in the comparative dataset. Towards this end, the activation of gene signatures from MSigDB C2 (literature curated) [12] were measured using the METABRIC dataset [13] in three distinct ways: using (1) patients with only *ER*-positive samples, (2) patients with only *ER*-negative samples, and (3) all patients. Figure 1a depicts the distribution of the absolute differences between the activation scores obtained by each tool using only *ER*-positive samples versus the scores obtained for all patients from METABRIC. This is repeated to compare *ER*-negative-specific scores versus all patients from METABRIC.

We observed a significant difference in the inferred activation score for all of the tested approaches (Wilcoxon’s *P* value < 0.0001 for all), with the largest differences observed for the *ER*-positive versus pan-METABRIC comparisons (Fig. 1a). Although the scores obtained from the four tools are incomparable, the results show that scores are influenced by perturbations in the patient composition of the dataset, establishing that none of these current approaches are absolute [9]. Analogous results were obtained when the dataset was stratified by *HER2* status and grade (Additional file 2: Figure S1), suggesting that many clinicopathological factors may influence pathway activation tools in this manner. Concretely, if those approaches were to be used in a clinical context, conclusions about the activation status of any given pathway would be greatly influenced by the comparative “control” group used by the treating clinician.

### A simple method to measure signature activation

The instability described above is analogous to what we and others have previously reported in the context of bioinformatics tools such as prediction analysis of microarray 50 (PAM50) that infer breast cancer subtype from gene expression profiles [9, 10]. In our previous work (AIMS), we developed a so-called absolute method that used only a gene expression profile from the target patient without recourse to or need of a larger compendium of patient profiles for comparative analysis [9]. Our goal here is to generalize and improve this framework to allow such absolute assignments to be made for any given molecular pathway or process for which there is a suitable gene signature.

The construction of our tool requires several components: (1) a collection of gene signatures that cover relevant biological processes including the hallmark molecular pathways/processes of breast cancer; (2) a suitably large collection of gene expression profiles of clinical breast cancer samples (ideally generated via both microarray and RNA-seq technologies) partitioned into training and validation datasets; and (3) a “gold standard” set of positive and negative patient profiles for each pathway with known activation status.

With respect to (1), we curated a collection of 6466 gene signatures from various databases of gene signatures including MSigDB [11] and GeneSigDB [14], which we complemented with our own in-house work to curate gene signatures especially applicable to breast cancer (*n* = 188, see “Methods” and Fig. 2a). With respect to (2), our training dataset comprised 4510 gene expression profiles obtained from 10 different cohorts on six different platforms (Table 1). We used our previously published dataset for validation (Affymetrix Gene ST, *n* = 429 patients [12]) and the most recent RNA-seq data from The Cancer Genome Atlas (TCGA) project (Table 1). We next describe how we estimate our gold standard.

### Estimation of the activation levels of each gene signature

Our goal is to train our AIPS algorithm to accurately infer the activation status of a given pathway within an expression profile of a patient. In order to train our algorithm, we require examples of patients that have activation statuses that we believe to be correct for each gene signature of interest. Furthermore, the examples must cover all possible states (e.g. high, low, and latent activation). The nature of human clinical samples, however, does not allow us to determine the activation status of a pathway in a direct, rigorous manner. Therefore our gold standard learning set must be comprised of estimations of statuses across the relevant expression datasets (item (2) as mentioned previously).

For each biological process of interest (item (1) as mentioned previously), we applied a *de novo* non-parametric rank-based method that partitions the patients in our dataset into three classes depending on the pattern of expression exhibited by the genes within the signature. The three classes correspond to those patients that appear to have high activation of the signature, low activation of the signature, and a set of patients where the expression of the genes within the signature lose their characteristic pattern of pairwise correlation (Fig. 1b provides an example). The latter class is assigned to patients where the corresponding gene expression patterns are pairwise independent, thus supporting neither high nor low activation of the underlying pathway.

This *de novo* non-parametric test, referred to as the ROI at quantile q (ROI_q_), proceeds as follows. In a univariate fashion, each gene within a given signature is used to rank all patients from the lowest to the highest expression. In some cases, the direction of expression of each gene within the signature is known *a priori* (e.g. the gene is overexpressed or underexpressed in samples with activation of the target pathway). Before ranking, we first negate any expression measurements for genes that are known to be underexpressed: such genes that are negatively correlated with activation of the signature, order the patients in the reverse order. Now for each patient, the ranks of all *k* genes from the signature are summed (see Additional file 1 for full details). The patients are then linearly ordered from the lowest to the highest rank. The approach of mapping expression data to a linear order, which has been used previously in breast cancer [15], makes intuitive sense as we can view the expression of each molecular process or pathway as having a state between “turned off” and “turned on” completely. Figure 1b depicts a proof-of-concept linear ordering for an estrogen response signature from Doane et al. [16] using the METABRIC dataset (Table 1). Broadly speaking, such linear orders highlight patients at the left hand side that have low or negative expression of the signature, patients at the right hand side that have high or positive expression of the signature, and a region in the middle corresponding to patients with gene expression patterns that are independent. We refer to this as the observed linear order.

The second step in the ROI_q_ procedure identifies the left and right boundaries of the low and high regions within the observed linear order. This is done via a permutation test where an “artificial” patient “n + 1” is created. Each of the *k* genes in the signature rank patient n + 1 with a uniformly randomly chosen number from (0… n + 1). Summing the randomized rank over all *k* genes in the signature, the position of patient n + 1 is computed within the observed linear order. This is repeated a suitably large number of times (e.g. *n* = 10,000). The ROI_q_ is defined as the region that contains *q*% of the randomly generated samples (Fig. 1b bottom and see Additional file 1).

As expected, the patient ordering at ROI_95_ for the estrogen response signature depicted in Fig. 1b is strongly associated with breast cancer subtype as defined by ER and HER2 status. In particular, the low activation region of the ordering (left) is enriched for ER-negative and/or HER2-positive tumors (Fisher’s exact test, *P* < 0.000001, Fig. 1b, c), whereas the high activation region corresponds almost exclusively to ER-positive/HER2-negative tumors (Fisher’s exact test, *P* < 0.000001, Fig. 1b, c). Given that ER-positive status is strongly associated with good outcome in breast cancer [12, 17, 18], the patient partition produced by the ROI_95_ is strongly prognostic (log-rank *P* < 0.0001, Fig. 1d). Although only a single proof of concept example, the results suggest that the ROI_q_ approach is capable of assigning pathway activation in breast cancer expression datasets. A more thorough investigation of the ROI_95_ is presented in Additional file 1. The analyses suggest that the ROI_95_ approach can faithfully recapitulate the low, independent, and high partitions of patients over a large range of biologically plausible parameters. For example, using simulated data, we tested the impact of several parameters including, for example, the gene signature size, fraction of patients in each category, and the strength of the signal on the capacity of the ROI_95_ approach to correctly assign patients in the low, independent, and high categories. We confirmed using this simulated set of data that the ROI_95_ is a robust approach within a wide range of parameters (see Additional file 1 and Additional file 2: Figure S2 and S3).

### Identification of informative and non-informative gene signatures

To better ensure that the ROI_95_ is accurately determining pathway status, we applied the method to all gene signatures in our collection (*n* = 6466, Fig. 2a) using the METABRIC dataset (*n* = 1992). The fraction of low, independent, and high samples across all signatures in our collection is presented in Fig. 2b. As a control, the gene labels of the METABRIC dataset were randomly permuted. This procedure should, with high probability, break the vast majority of gene-gene correlations within signatures, causing the fraction of uninformative genes to rise. We should then observe an increase in the independent partition of the ROI_95_ with a concomitant decrease in the size of the low and high partitions. The results depicted in Fig. 2b confirm this, and suggest that for a large proportion of the signatures, the ROI_95_ method is indeed assigning activation status in a very non-random fashion.

The results depicted in Fig. 2b also suggest that the ROI_95_ method assigned almost every patient to the intermediate partition for some signatures. In other words, the ROI_95_ method applied to these specific gene signatures was not distinguishable from random expression patterns. We removed all such gene signatures from further consideration, in particular, a gene signature was removed when the fraction of samples in the ROI_95_ region exceed 0.8, as this is no better than partitions generated by random sampling. This led to a list of 3472 signatures that we considered informative in the context of breast cancer. A cutoff on the ROI_95_ region will exclude gene signatures activated or repressed in less than 20% of samples. Although more liberal thresholds could be used when studying an individual gene signature, we chose this conservative threshold here to enable our high-throughput global analyses.

Given that our gene signatures were collected from various sources, we asked whether any particular source was enriched for uninformative signatures. Of the remaining informative signatures, pathway databases such as BioCarta, Kyoto Encyclopedia of genes and genomes (KEGG) and Gene Ontology (GO) have higher fractions of signatures that have near random behavior (bottom of Fig. 2c). We note that sources that contributing signatures from transcriptional profiling have a higher proportion of non-random signatures (top of Fig. 2c).

### Absolute single-sample gene signature activation in breast cancer

Based on the aforementioned results, we used the ROI_95_ method with the 3472 informative signatures to the training and validation datasets for calling signature activation levels using only the expression profile of a given single patient. The approach used here broadly follows our AIMS method that infers breast cancer subtype (Fig. 3a; also [9]).

First, the ROI_95_ is applied to each informative signature across 10 expression datasets generated from several microarrays (one-color and two-color) and RNA-seq platforms totaling 4510 samples (Table 1). This large and diverse training dataset provides us with more confidence that biases for specific clinicopathological or other patient variables are ablated, or at least reduced [9]. Our learning set consists of activation statuses (low, independent, and high) for 3472 signatures in each of 4510 patients from the training set.

Now, for each signature a model is learnt as follows. First, we consider every possible pair of genes *x* and *y* in the expression dataset, and we ask how strongly the rule “if expression of *x* is greater than *y*, then classify signature as high” is associated with our gold standard learning set. This is repeated for a modification of the rule where *x* is less than *y*, and for the other activation statuses of independent and low. Then, for each signature, the *K* most highly associated rules are chosen from the ranked list of all possible gene pair rules. The *K* rules are combined into a single probabilistic model using a naive Bayes’ classifier, and validated on an independent dataset (*n* = 742 samples, Table 1) [12].

The last step of our approach consists in selecting only those models with strong agreement with the ROI_95_ approach using a cutoff of 0.05 on the Bonferroni adjusted Kappa’s statistics *P* value. The two filtering steps that consist of first filtering out non-informative gene sets and then keeping models with significant agreement are essential to provide a set of reliable models (Fig. 3a).

### Approximately 50% of informative signatures are amenable to absolute assignment

To ensure that our models are applicable across different technologies, we only retained models that significantly agreed with the gold standard in all 10 of the training datasets (kappa statistics Bonferroni-adjusted *P* value <0.05). This resulted in the retention of 1733 models (1733/3472, approximately 50%). We observed that the retained models had better agreement with the gold standard in the validation dataset in comparison with the models that were removed (Fig. 3b). This observation suggests that our training procedure did not introduce any significant over-fitting as selected models behave similarly in the training and validation sets and also all the models obtain a significant kappa statistic in the validation set (kappa statistics *P* values <0.01 for all).

We also stress here that the validation dataset contains data generated on a microarray platform (Affymetrix Gene ST) not present in the training dataset, suggesting that AIPS assignments are applicable on technologies not utilized in the training procedure. AIPS correctly assigns activation status for samples either assigned low or high activation (mostly red (approximately 80%) for the low-low and high-high lines in Fig. 3c). About 60% of samples were assigned to the independent class over all the 1773 models,

For the samples in the gold standard that were assigned independent status, AIPS correctly assigned this status in 60% of the cases, suggesting that predictions made for samples in the independent class are generally less reliable than predictions made for the low or high classes (mostly yellow for the “ind.-ind.” tagged line in Fig. 3c). Importantly, we rarely observed cases were AIPS predicted high activation when the gold standard was low, and vice versa (mostly blue for the low-high and high lines in Fig. 3c).

Last, we note that the AIPS models used fewer genes to infer pathway activation status than the original ROI_95_ method to generate the gold standard (median 50 versus 200 genes for AIPS versus ROI_95_ respectively; Wilcoxon’s test *P* < 0.0001, Fig. 3d).

Overall, these analyses confirmed that AIPS could accurately recapitulate the assignments of the gold standard. The 1733 AIPS models are listed in Additional file 3: Table S1 and pathway activation assignments can be computed for new individual samples using our AIPS R package [19].

### Absolute assignment of *EGFR* pathway activation using AIPS

Epidermal growth factor receptor (*EGFR*) is well-studied in breast cancer with high activation of this pathway associated with poor patient outcome [20, 21]. We examined the behavior of our AIPS-*EGFR* model in the McGill validation dataset (Fig. 3e–g). We observed that the activation of samples at the far left and right (low and high respectively) are nearly perfectly inferred by AIPS (kappa = 0.54, *P* < 0.0001) with the majority of disagreements related to samples in the independent region (Fig. 3e–g). Figure 3f depicts the simple binary rules used by the AIPS model for the *EGFR* signature across the patient samples. There is a large cluster of *EGFR*-high patients associated with the PAM50 basal-like (BasalL) subtype, and a second large cluster of EGFR-low patients associated with luminal A and luminal B subtypes, a finding consistent with previous studies [20, 21].

Interestingly, gene set enrichment analysis of the genes selected to participate in the binary rules revealed an enrichment for genes upregulated by *EGFR* in MCF7 cells (FDR *q* value = 1.45e-17) [22]. Furthermore, all of these genes are on the right side (or left side) of binary rules associated (or not-associated) with high EGFR activation (rules marked by an asterisk in the heatmap of Fig. 3f). Although AIPS selects gene pair rules for each model from the large space of all possible gene pairs, it still surprisingly often selects genes that were present in the original signature, and therefore are likely good markers of the underlying biological processes. The enrichment of genes from the original signature was also reported for other “absolute” models such as AIMS [9]. Almost all of our AIPS models had such enrichment (1335 out of 1733 models (77%), Additional file 2: Figure S5). It is important to note that although most models statistically significantly overlapped the original signature, the number of genes from the original signature was still below 10%, suggesting that AIPS models do require many other genes to mimic the ROI95 assignments.

We asked if the absolute nature of the AIPS method would result in a more consistent *EGFR* model across gene expression platforms. In particular, we asked if our AIPS model inferred the same activation status for the EGFR pathway in both the microarray and RNA-seq platform for the same patient. Using TCGA data for 398 patients [23], AIPS assignments agreed on 87% of patients between both platforms (Fig. 3h, kappa = 0.81, *P* < 0.0001). Systematic analysis over the entire partitions (*n* = 1733 models) revealed that this agreement value is representative of almost all the partitions induced by AIPS and is significantly different from a random distribution (Fig. 3i, Wilcoxon’s test *P* < 0.0001, all kappa statistics *P* < 0.0001) supporting the argument that absolute assignments are robust across multiple platforms [9]. Together these results suggest that AIPS is capable of inferring signature activation levels with comparable performance to relativistic tools but with the added benefits of an absolute single sample approach.

### AIPS assignments agree with whole-cohort inferred pathway scores

**Fig. 4:**
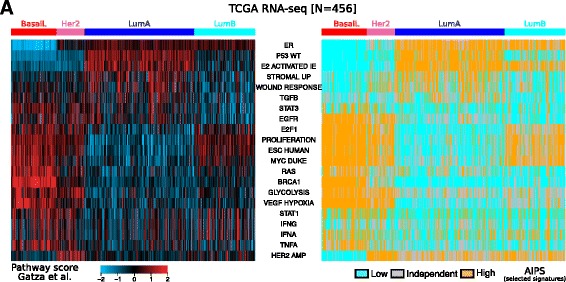
Assignment by absolute inference of patient signatures (*AIPS*) agrees with whole-cohort pathway scoring tools. **a** Comparison between the pathway scores retrieved from Gatza et al. and AIPS. The ordering of the *rows* and *columns* is preserved between the two heatmaps. *BasalL*, basal-like, *HER2* human epidermal growth factor receptor 2, *LumA* luminal A, *LumB* luminal B, *ER* estrogen receptor, *TGFB* transforming growth factor beta, *EGFR* epidermal growth factor receptor, *VEGF* vascular endothelial growth factor, *IFN* interferon

Our goal was to compare AIPS assignments with a second approach from the literature that takes full advantage of an entire dataset to assign signature activation scores. In particular, we used 21 non-redundant scores from the publication of Gatza et al. generated from breast cancer RNA-seq expression data from the TCGA project (*n* = 456) [24]. Concomitantly, we estimated activation status using our AIPS models for these signatures on the same patients. Overall good agreement between AIPS and the pathway scores from Gatza et al. was observed (Fig. 4a) although the two approaches are quite dissimilar.

Figure 4a suggests that well-known breast cancer biological processes are recapitulated by AIPS assignments. For example, patients with the luminal A or B subtype (LumA or LumB) are mostly assigned to the AIPS-high class for the *ER* gene signature, consistent with the fact that subtypes are enriched form *ER*-positive patients [9, 25]. Also, the AIPS assignments are in good agreement with the proliferation, ESC human and *MYC* DUKE pathway scores as these processes are known to be associated with the highly proliferative basal-like (BasalL), *Her2* and LumB subtypes [25, 26]. We also observed a significant proportion of *Her2*-positive patients assigned to the AIPS-high *Her2* gene signature. The interferon alpha and gamma, *STAT1* and *TNF* alpha pathway scores are in good agreement with the AIPS assignments; these processes are associated with the BasalL subtype [27]. The *P53* WT signature from AIPS is in good agreement with the pathway scores and is enriched for the LumA subtype that has been shown to be depleted of *P53* mutations [23].

Generally, if AIPS modules and the pathway scores of Gatza et al. are in good agreement, then the patients within the high class of AIPS should also have the highest pathway scores according to Gatza et al. We tested the agreement between these two approaches and observed a strong relationship (Wilcoxon’s *P* < 1.4e-14 for all, Additional file 2: Figure S4A). Overall this analysis suggests that the “single-sample” AIPS approach is in good agreement with an approach that uses an entire cohort of samples to judge activation.

### Sample partitions induced by AIPS are prognostic and associated with breast cancer subtypes

As there are many pathways and process that are known to vary in their expression across breast cancer subtypes, we investigated the relationship between patient subtype (called using the AIMS tool [9] or using clinical information) and the entire patient partitions generated by AIPS on the McGill dataset (Table 1).

**Fig. 5:**
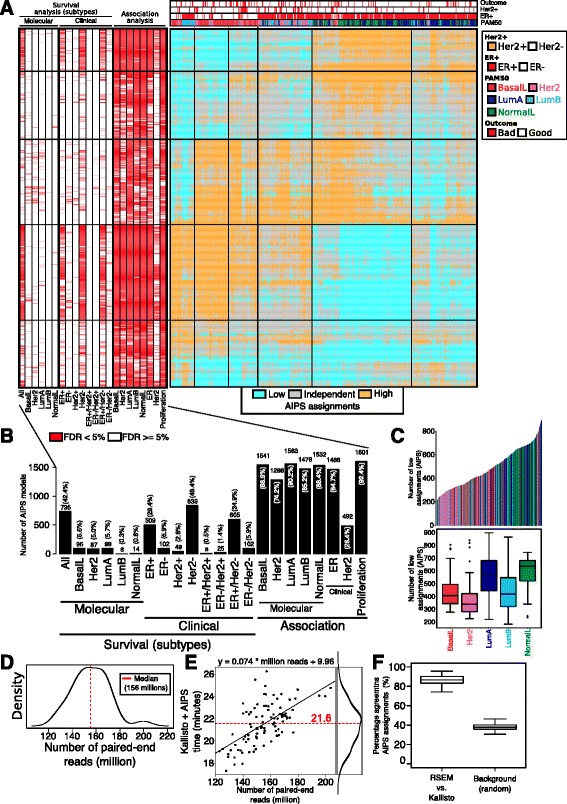
Absolute inference of patient signatures (AIPS) models are prognostic. **a** *Right* heatmap depicts the global partitions made by AIPS on the McGill validation dataset (*n* = 429, *cyan* low, *gray* independent, *orange* high). *Left* significant associations (false discovery rate (FDR) <5%) induced from AIPS partitions. *Far left* associations with survival (log-rank test) using the molecular and clinical subtypes and *far right* significant overlap with the clinical and proliferation molecular subtypes (Fisher’s exact test). **b** Presentation of the numbers and percentages of AIPS partitions that are significantly (FDR <5%) associated with either survival or overlap. **c** *Top* each bar represents the number of times a patient in the McGill validation dataset was assigned to the low class across all 1733 AIPS partitions. *Color* indicates the subtype of the patient. *Bottom* distribution of number of times patients of each subtype were assigned to the low class using AIPS partitions. **d** Distribution of the number of 100-bp paired-end reads use in the Kallisto quantification (*n* = 96). **e** Time taken in minutes for Kallisto quantification and AIPS partitions as a function of the number of paired-end reads. *Line* represents a linear fit of the data. **f** Kallisto versus RSEM AIPS partitions agreement versus a background distribution agreement obtained from randomly shuffling the labels 100 times. *BasalL*, basal-like, *HER2* human epidermal growth factor receptor 2, *LumA* luminal A, *LumB* luminal B, *ER* estrogen receptor, *NormalL* normal-like

We first studied the relationship between the partitions induced by AIPS and survival for the different molecular and clinical subtypes (Fig. 5a, b). We noticed that almost half (42.4%) of the partitions are significantly associated with survival if the analysis is performed on the entire cohort (Fig. 5a, b; Additional file 3: Table S1). This number drops drastically if we restrict the survival analysis to patients of given subtype. For example, only around 5% of the partitions are significantly associated with survival for the BasalL, *Her2* and Luminal A and to close to nothing for the LumB and normal-like (NormalL) subtypes. Similarly, for the clinical subtypes, we found between 30 and 50% of partitions associated with the *ER*-positve, *Her2*-negative and *ER*-positive/*Her2*-negative subtypes. Those numbers drops between 3 and 6% for the *ER*-negative, *Her2*-positive and *ER*-negative/*Her2*-negative subtypes. We found almost no partitions associated with the *ER*-positive/*Her2*-positive and *ER*-negative/*Her2*-positive clinical subtypes (Fig. 5a, b; Additional file 3: Table S1).

We also studied the association between AIPS partitions and the molecular, *ER*, *Her2* and proliferation score [25] using Fisher’s exact test (Fig. 5a, b). We found that between 85 and 92% of the partitions are associated with the different grouping with the exception of the Her2 subtype and the clinical features (Fig. 5a, b). Almost all (92.4%) partitions are associated with proliferation.

We also examined the frequency of patients classified as low over the entire set of 1733 AIPS models (Fig. 5c). Patients with the LumA and NormalL subtyptes obtained a significantly higher number of assignments compared to the those with the remaining subtypes, with an increase of between 200 and 250 in low assignments (Fig. 5c).

### AIPS applied on single RNA-seq samples can be performed in a timely fashion

AIPS should enable a significant amount of information to be extracted from single RNA-seq samples and the growing number of single-cell sequencing datasets. We measured the time necessary to obtain AIPS partitions from a single RNA-seq profile using Kallisto, a fast pseudoalignment program used to obtain transcript quantification from sequencing data [28].

Using 100-bp paired-end sequencing data (median of 155 million paired-ends per patient [28], *n* = 96, Fig. 5d) we monitored the time taken to obtain transcript quantification and AIPS partitions using a single central processing unit (CPU). Overall, it required a median 21.6 minutes to obtain AIPS partitions for an individual patient directly from raw paired-end sequencing data (Fig. 5e).

Given that different transcript quantification pipelines return slightly different results, we evaluated the agreement between AIPS partitions made using Kallisto quantification to partitions made using an alternative approach (Bowtie2 [29] + RSEM [30]). Overall, we found significant agreement between AIPS partitions made from the two quantification approaches (Wilcoxon’s test *P* < 0.0001 and all kappa statistics *P* < 0.0001) with a median agreement of approximately 85% (Fig. 5f). Together this establishes that AIPS could be applied in a time-effective manner on single-sample RNA-seq data with the aid of a sufficiently fast pseudoalignment program e.g. Kallisto.

## Discussion

The work presented here is predicated upon the observation that existing pathway analysis tools are relativistic in nature. In essence, the tools make use of a large panel of samples to “judge” whether there is evidence that the given pathway is underexpressed or overexpressed relative to the panel. We showed here that the scores returned by these tools are sensitive to the composition of patients within the dataset, using a large breast cancer gene expression dataset. More precisely, we showed that the scores returned by these tools vary when the frequency of specific clinicopathological variables is perturbed. Although we have shown this is true for grade, *ER* and *HER2* status, it is likely that many other variables that were assessed (e.g. age, claudin-low status, tumor size) and or not assessed (e.g. tumoral heterogeneity, clonal complexity, lifestyle and information on exposure of the patient) can also affect the estimations of pathway activity using these tools [31–33]. This is non-unintuitive: if the gene expression profile of a patient is included in two different datasets with marked differences in the overall composition of patients (e.g. they differ on the fraction of *ER*-positive patients), in one dataset a target pathway could be assessed as having high activation but in the second dataset as low. As these, or similar variables, are involved in most, if not all cancers, it is highly likely that this degenerate behavior is not restricted to studies of breast carcinoma. Clearly an “absolute” tool that ablates this “relativistic” behavior would be a step in the right direction.

The main focus of this study is the development of a *de novo* framework to estimate the activity of a given pathway using only a single sample. Here we have trained and validated predictive models for 1733 gene signatures for these pathways using a large compendium of breast cancer gene expression profiles. The profiles originate from several distinct microarray and RNA-seq platforms. In order to develop a suitable training and validation dataset, we developed the notion of the *q*% region of independence (ROI_q_), which assigns simple discrete levels of activation for a given gene signature and a sufficiently large dataset. Using a large number of synthetic datasets we have shown that the method is robust and can faithfully retrieve low and high activation for many gene signatures within realistic configurations (see Additional file 1). By extending our previous AIMS methodology [9] with this gold standard, we were able to generate 3472 absolute single-sample gene signature activation models of which approximately 50% (1733/3472) performed sufficiently well as to be included in AIPS. We have shown that the AIPS models are more compact, their assignments are reproducible when the same patient is profiled using two distinct platforms, and the models are highly prognostic. Moreover we showed that our ability to estimate the activation of most pathways is not reduced when switching from a relativistic to an absolute method.

AIPS provides 1733 models that are immediately applicable to new breast cancer samples even when they are profiled in isolation, outside of a large cohort to make comparative assessments. Furthermore, we have shown that AIPS models are prognostic and compare favorably to other whole-cohort approaches, and that AIPS could be applied effectively to RNA-seq data. The term “absolute” expresses the idea that pathway assessment made on such a new “isolated” sample is a function only of the learning phase for each of the 1733 models, and is not done relative to a comparative cohort. This is, to the best of our knowledge, a marked difference from all other pathway tools currently available. The power of this approach is that it allows us to completely define and control the learning set, removing biases and potential confounding variables in downstream analysis. This is not possible with other current approaches where pathway analyses are affected by the other patient profiles in the cohort. Of course, here “absolute” does not imply that pathway assessments are perfect nor can the method judge in all cases the state of a pathway in a tumor relative to a healthy normal control. However, the presence of normal-like samples in our training sets allows us to assess such an “absolute state” of the pathway (the AIPS R package includes this analysis). Of course, the quality and definition of the AIPS models is still a function of the training set. In other words, modifications to the training set might impact model definitions, but here we trained our models over multiple large cohorts in order to minimize the risk that small specialized datasets would skew model parameters. Further refinement and curation of the learning dataset might potentially lead to absolute models.

Although there is a steadily increasing number of breast cancer gene signatures derived from microarray and RNA-seq based studies, at best a dozen of these signatures are currently directly available to clinicians and patients [34, 35] and almost all of them are suspected to be essentially sophisticated multigene predictors of proliferation [12, 26, 36, 37]. Given an expression profile of a patient sample, AIPS is able to estimate the activation of 1733 of the pathways, molecular processes and functions simultaneously in a timely fashion. This represents a step towards a clinically feasible tool that would provide healthcare providers and clinicians with important information on many aspects of the tumor beyond proliferation.

## Conclusions

Kim et al. [38] recently reiterated our observation of the relativistic nature of all current clinical gene expression-based prognostic tests and acknowledge the problematic nature of this situation. The authors suggest that an absolute method could be used with RNA sequencing data to robustly identify patients with a luminal A subtype that may not need chemotherapy in a manner analogous to Oncotype Dx [39]. AIPS represents such a solution and our analyses here suggest that 70% of models (1203/1733) are able to distinguish patients with luminal A cancer from patients with luminal B cancer (FDR <0.01 (Fisher’s exact test), Additional file 3: Table S1). This will provide a broad range of molecular pathways and processes to develop an absolute clinical test to measure patient benefit from chemotherapy.

## Additional files

Additional file 1: Supplemental methods. (PDF 589 kb)

Additional file 2: Figure S1. Instability of current pathway activation tools in function of grade and Her2. **Figure S2.** The ROIq method is able to identify samples with either low or high activation. **Figure S3**. The ROIq method is able to identify samples with either low or high activation. **Figure S4.** Comparing pathway scores from Gatza et al. to AIPS assignments. **Figure S5.** Comparing genes selected in AIPS models with genes in the original gene signature. (PDF 782 kb)

Additional file 3: Table S1. Information on the 1733 selected AIPS models. (XLS 3145 kb)

## Abbreviations

AIMS: Absolute intrinsic molecular subtyping
AIPS: Absolute inference of patient signatures
BasalL: Basal-like
BH: Benjamini–Hochberg
bp: Base pair(s)
CDF: Cumulative distribution function
DART: Diversity arrays technology
EGFR: Epidermal growth factor receptor
ER: Estrogen receptor
FAIME: Functional analysis of individual microarray expression
FDR: False discovery rate
GSVA: Gene set variation analysis
HER2: Human epidermal growth factor receptor 2
IQR: Interquartile range
LumA: Luminal A
LumB: Luminal B
METABRIC: Molecular Taxonomy of Breast Cancer International Consortium
NormalL: Normal-like
PAM50: prediction analysis of microarray 50
PLAGE: Pathway level analysis of gene expression
RNA-seq: RNA sequencing
ROI: Region of independence
ROIq: Region of independence at quantile q
ssGSEA: Single-sample gene set enrichment analysis
TNF: Tumor necrosis factor
