# Erratum to: Detecting gene signature activation in breast cancer in an absolute, single-patient manner

**DOI:** 10.1186/s13058-017-0842-5

**Published:** 2017-04-13

**Authors:** E. R. Paquet, R. Lesurf, A. Tofigh, V. Dumeaux, M. T. Hallett

**Affiliations:** 1 Centre for Bioinformatics, McGill University, Montreal, Quebec H3G 0B1 Canada; 2 The Rosalind and Morris Goodman Cancer Research Centre, McGill University, Montreal, Quebec H3A 1A3 Canada; 3 School of Computer Science, McGill University, Montreal, Quebec H3A 0E9 Canada

## Erratum

This article [1] has been updated to correct an abbreviation. DART is an abbreviation of ‘Denoising Algorithm using Relevance network Topology’ and not ‘Diversity arrays technology (DART)’, as previously stated in the article. The abbreviation has been changed in two places; In the abbreviations section and in the first paragraph of the backgrounds section.
