# Supplementary material for: Detecting gene signature activation in breast cancer in an absolute, single-patient manner
Source: Breast Cancer Res. 2017 Mar 21;19:32. doi: 10.1186/s13058-017-0824-7 (PMC5361722; doi:10.1186/s13058-017-0824-7)
Supplement: Supplementary file 1 — Supplemental methods. (PDF 589 kb) [file 13058_2017_824_MOESM1_ESM.pdf]

Supplemental methods

Detecting gene signature activation in breast cancer in an absolute, single patient manner

Paquet ER[1,2,3*], Lesurf R[1,2,3*], Tofigh A[1-4], Dumeaux V[1], and Hallett MT[1-4]

Linear ordering of patients

Our approach is to map samples to a linear ordering based on expression of the selected features within a given signature. This is in contrast to more generalized approaches that map samples to tree metrics such as hierarchical clustering. The intuition for this restriction to a linear ordering is that the activity of many individual processes is well modeled by a simple continuous score (eg. activity ranging from 0 to 1). Several distinct algorithmic and statistical approaches to linear orders are possible but we use here a simple ranked-based method described as follows.

For a given gene expression D with m genes and n samples, let $D(g,p)$ be the observed expression of gene g in sample p. For a signature S, let S^+ be the subset of genes in S that belong to the positive (overexpressed) partition, and let S^- be those that belong to the negative partition. For a signature S and dataset D, we define the corresponding rank matrix R as

$$R(g,p) = \begin{cases} \left|\{p' : D(g,p') \le D(g,p)\}\right| if\, g \in S^+, \\ \left|\{p' : D(g,p') \ge D(g,p)\}\right| if\, g \in S^-. \end{cases}$$

For a sample p in D, we define the rank statistic $\rho(p)$ as the sum of the ranks in R:

$$\rho(p) = \sum_{g \in S} R(g,p).$$

The linear order π of the samples based on the genes in S is the order induced by ρ. We use r to denote the rank of a patient in this ordering:

$$r(p) = \left|\{p' : \rho(p') \leq \rho(p)\}\right|.$$

When necessary, we will use subscripts to make explicit the dataset D and signature S and will refer to the above entities as: $R_{D,S}$, $\rho_{D,S}, \pi_{D,S}, r_{D,S}$.

Region of Independence

For a signature S and dataset D, we are interested in identifying those patients that have either notably low or high activity of the process represented by the signature S. The samples with the lowest ranks represent samples that exhibit the least activity, while those with the highest ranks represent samples with the highest activity. Our goal is to identify values for L and $H, 0 \leq L \leq H \leq n+1$ so that samples with rank ≤ L represent patients with a significant decrease in the activity represented by the signature S and patients with rank ≥ H represent a significant increase in activity. We do this by means of a random sampling procedure, described below, that examines the strength of correlation between pairs of genes in S along the patient ordering induced by $\rho_{D,S}$.

With respect to a signature S, we can distinguish three extreme types of samples:

1. Samples whose expression of genes in S^{+} are positively correlated and greater than average, while the reverse is true for the expression of genes in S^{-}. These samples will have high rank values $r_{D,S}$.
2. Samples whose expression of genes in S are independent and do not exhibit the correlation structure indicated by the partition of S into S^{+} and S^{-}.
3. Samples whose expression of genes in S+ are positively correlated and lower than average, while the reverse is true for the expression of genes in S^{-}. These samples will have low rank values in $r_{D,S}$.

We propose the following random sampling procedure for partitioning the samples into three parts corresponding to the above three types. We first extend the rank matrix R with a new column n + 1 with values drawn independently from a uniform distribution:

$$R(g, n + 1) \sim U(0, n + 1), g \in S.$$

Let R' be the rank matrix obtained by reranking the rows in the extended R and let ρ′ and r′ be defined as before, but computed based on R' instead of R:

$$\rho'(p) = \sum_{g \in S} R'(g, p),$$

$$r'(p) = |\{p' : \rho'(p') \leq \rho'(p)|.$$

where p = 1...n + 1. The rank r′(n + 1) corresponds to the rank of a new sample with independent gene expression values. We perform the above random sampling procedure many times to obtain a distribution of ranks from r′(n+1) and define L and H as L = a − 1 and H = b, where a and b are the α/2 and 1 − α/2 percentiles of the distribution of the ranks. Together, L and H define what we call the (1 − α)-region of independence, or $ROI_{1-\alpha}$. Unless stated otherwise, we use a sample size of 10000 and α = 0.05.

Missing values

A recurring feature of gene expression experiments is missing values. If only a relatively small number of genes in a signature have missing values, one solution is to simply remove such genes from the analysis. When this is infeasible or otherwise undesirable, we use an alternative version of our rank statistic based on normalized ranks as follows. We define the rank matrix for a signature S as

$$R(g,p) = \begin{cases} 0 & \text{if } D(g,p) \text{ is missing,} \\ \dfrac{\left|\left\{p' \in P_g : D(g,p') \le D(g,p)\right\}\right|}{\left|P_g\right|} & \text{if g} \in \text{S}^{+}, \\ \dfrac{\left|\left\{p' \in P_g : D(g,p') \ge D(g,p)\right\}\right|}{\left|P_g\right|} & \text{if g} \in \text{S}^{-}. \end{cases}$$

where P_g is the set of samples for which *D(p, g)* is not missing. The rank statistic ρ will now be the average of the normalized ranks in R:

$$\rho(p) = \frac{\sum_{g \in S} R(g,p)}{\left|\left\{g \in S : D(g,p) \text{ is not missing}\right\}\right|}.$$

Finally, the rank of each patient in D with respect to S is defined just as before:

$$r(p) = \left|\left\{p' : \rho(p') \leq \rho(p)\right\}\right|.$$

Synthetic dataset generation

The generation of a synthetic dataset to test the performance of the ROI can be defined as a function of five parameters: 1) k : the number of genes in a gene signature 2) n : the number of samples in the dataset 3) the fraction of low (f_l) and high (f_h) patients 4) ı : the fraction of informative genes in signature, and 5) μ: the gene signature effective signal. The first step consists in defining a matrix $M[g_i,p_j]$ where g_i corresponds to gene i and p_j corresponds to sample j. The size of this matrix is controlled by the parameters n and k. We initialize this matrix by sampling values from a normal N(0,1) distribution. The second step necessitate the definition of two subgroups of patients S_{low} and S_{high} that will correspond to indices of patients assigned to the low and high activation:

$$S_{low} = [1 \ldots (f_l{*}n)]$$

$$S_{high} = [(n - f_h{*}n) \ldots n].$$

We also need to define a list of indices for the informative genes G_{cons}:

$$G_{cons} = [1 \ldots (k{*})].$$

Once we have the informative list of genes G_{cons} and the list of low (S_{low}) and high (S_{high}) samples we can impute the final gene signature signal to matrix M like this:

$$M[G_{cons},S_{low}] \sim N(-\mu,1)$$

$$M[G_{cons},S_{high}] \sim N(\mu,1)$$

The matrix M is now ready to be utilized to test the ROI_{95} approach by testing every combinations of the five pairs of parameters and generating 20 different random dataset per combinations of parameters to get precise estimate of the ROI_{95} performance using a given set of parameters. When a parameter is not one of the two that are varied in a particular experiment, the default values listed in Supplementary Table S2 are used. We measure the agreement between the ROI_{95} and the ground truth low, independent and high assignments in the synthetic dataset using the average agreements for the low, independent and high assignments obtained from the ROI_{95}. Briefly, suppose A_l corresponds to the percentage of patients assigned low by the ROI_{95} that are also assigned low in the synthetic dataset, A_h and A_I correspond to the same definition except for the ROI_{95} assigned high and independent patients. The metric we used to assess the overall agreement of the ROI approach is:

$$(A_l + A_l + A_h)/3$$

This metric has the advantage of giving equal weights to the three classes of patients. We also tested the significance of the agreement and marked it using asterisks in Additional file 2: Figure S2 and S3 using the Cohen's *kappa* statistics implemented in the "fmsb" R package.

The $\mathbf{ROI_q}$ method is able to identify samples with either low or high activation

To test the robustness of this new approach for constructing a suitable learning set, we used a large panel of synthetic datasets (~N=40,000) that covered the range of parameter values that the ROI_q would be confronted

when presented with real experimental data. For example, in our synthetic datasets we can control the percentages and identity of the low, independent and high patients, allow us to mimic the percentages observed in the real experimental data used in the ROI_q approach (Additional file 2: Figure S2A).

Repeated permutation testing was used with six central parameters. The number of samples in the dataset (n) was varied in our simulations to mimic the fact that the range in size of breast cancer datasets varies (see for example Table 1 in the main text). Since signatures come from many different sources and cover a diverse range of biologies, we varied the number of genes of the signature (k). In any given dataset, we would expect that there is a natural variation in the number of samples that have high activation, low activation or independence with respect to any given biological process. To investigate this, differences in the size of the low, high and independent regions $f=(f_l, f_i, f_h)$ were explored (Supplementary Table S2 in this document). We and others have observed that some genes in almost any given signature, and in any given dataset, appear uninformative(1,2). This may be expected given that such signatures were likely learnt on a different platform, perhaps via different experimental systems (eg. mouse models, cell lines), and within a range of dataset sizes with varied clinico-pathological attributes. To investigate this, we considered a parameter (ı) that controls the fraction of genes in a signature that are informative; that is, the genes show consistent differential expression in either the low, independent or high partitions. (Additional file 2: Figure S2A and Supplementary Table S2 in this document). We assume that the expression of informative genes in the signature are

distributed according to N(μ, 1) and N(-μ,1) for samples in the high and low class respectively. The variable allows us a simple way to adjust the effect size (Additional file 2: Figure S2A; Supplementary Table S2 in this document). The distribution of expression for all genes in the signature for samples within the ROI are distributed according to N(0,1). This distribution is also used for all non-informative genes from samples within the low and high classes. In total, the system is defined by five parameters: n, k, f, ι, μ.

Multiple synthetic datasets (n=20) were generated after varying exactly two (of five) parameters simultaneously (Supplementary Table S2 in this document). The ROI_{95} method was then applied to each dataset, and the resultant low, independent and high partition was compared to the artificially synthesized ground truth (Additional file 2: Figure S2B-E and S3A-F). For a large number of configurations, we observed significant agreements between the estimated ROI_{95} and the synthetic ground truth. In Additional file 2: Figure S2B, when the fraction of informative genes was at least 0.7 and the mean of the effect at only 0.7 (variance 1), 95% of the low, independent, high partitions are retrieved. In Additional file 2: Figure S2C, focusing again on samples that were greater than 0.7 as the fraction of informative genes, the ROI_{95} method recuperates over 90% of the sample partition when the overall size of the signature is greater than 70 genes. We note that 3946 of the signatures in our compendium used here have > 70 genes. We observe a large range of behaviors when the frequencies $f = (f_l, f_i, f_r)$ are varied (Additional file 2: Figure S2D). If we focus on trials that were at least 0.7 informative (ι), we observe that the performance of the method decreases as the size of the

independence region (f_i) decreases (ie. the samples are more evenly spread across all three partitions). This is likely due to the fact that the ROI_{95} method requires a dataset with enough samples in the low and high partitions. When there are too few, the permutation testing fails to accurately define the left and right boundaries of the independent region. The bottom of Additional file 2: Figure S2D, highlighted in blue, presents configurations with different percentages of low and high patients. We observe that the ROI has slightly better agreements when the fraction of low and high samples are approximately equal (bottom Additional file 2: Figure S2D). Surprisingly, the number of samples n in the dataset does not have a substantial effect on performance, and this is especially true for dataset sizes observed in the literature where n most exceeds 100 (Additional file 2: Figure S2E and Table 1 in the main text). Overall, these analyses suggest that the ROI_{95} approach can faithfully recapitulate the low, independent and high partitions over a large range of biologically plausible parameters (see also Additional file 2: Figure S3A-F).

Supplementary Table S2. Parameters used in the evaluation of the region of independence (ROI_{95}) approach. In any experiment, at most two parameters are perturbed simultaneously. Default values for non-perturbed parameters are denoted in brackets.

**(1) Number of genes in signature (k) [50]**	**(2) Number of samples n [200]**	**(3) Fraction (low,independent,high) fl fh[0.1,0.8,0.1]**	**(4) Fraction informative ι [.8]**	**(5) μ [.5]**
10	10	0.05,0.9,0.05	0.1	0.05
20	20	0.1,0.8,0.1	0.2	0.1
30	30	0.2,0.6,0.2	0.3	0.2
40	40	0.3,0.4,0.3	0.4	0.3
50	50	0.4,0.2,0.4	0.5	0.4
60	60	0.45,0.1,0.45	0.6	0.5
70	70	0.05,0.45,0.5	0.7	0.6
80	80	0.1,0.45,0.45	0.8	0.7
90	90	0.2,0.4,0.4	0.9	0.8
100	100	0.3,0.35,0.35	1	0.9
200	150	0.4,0.3,0.3		1
300	200	0.5,0.25,0.25		1.5
500	250	0.6,0.2,0.2		2
1000	300	0.7,0.15,0.15		
	350	0.8,0.1,0.1		
	400	0.9,0.05,0.05		
	450			
	500			

References

1. Hanzelmann, S., Castelo, R. and Guinney, J. (2013) GSVA: gene set variation analysis for microarray and RNA-seq data. *BMC Bioinformatics*, **14**, 7.
2. Tomfohr, J., Lu, J. and Kepler, T.B. (2005) Pathway level analysis of gene expression using singular value decomposition. *BMC bioinformatics*, **6**, 225.
