# Supplementary material for: Detecting gene signature activation in breast cancer in an absolute, single-patient manner
Source: Breast Cancer Res. 2017 Mar 21;19:32. doi: 10.1186/s13058-017-0824-7 (PMC5361722; doi:10.1186/s13058-017-0824-7)
Supplement: Supplementary file 2 — Instability of current pathway activation tools in function of grade and Her2. Figure S2. The ROIq method is able to identify samples with either low or high activation. Figure S3. The ROIq method is able to identify samples with either low or high activation. Figure S4. Comparing pathway scores from Gatza et al. to AIPS assignments. Figure S5. Comparing genes selected in AIPS models with genes in the original gene signature. (PDF 782 kb) [file 13058_2017_824_MOESM2_ESM.pdf]

Supp. Fig. 1. Paquet et al.

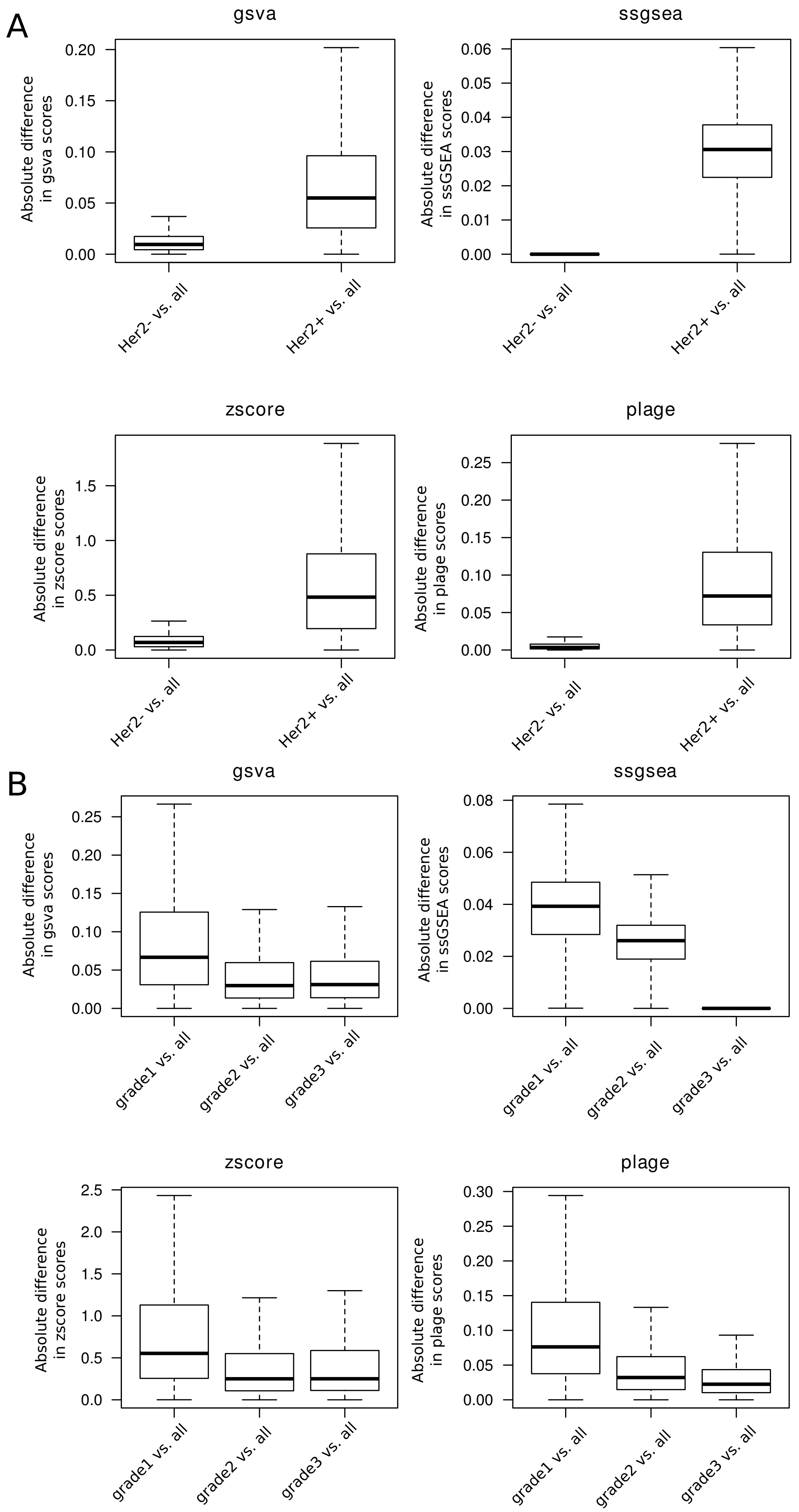

Supplementary Figure S1. Instability of current pathway activation tools in function of grade and Her2 (A) Boxplots represent the absolute difference between the score obtained from a specific pathway activation tool (GSVA, ssGSEA, zscore and plage) using all patients from the METABRIC dataset, and the score obtained when METABRIC is restricted to either Her2+ samples (left) or Her2- samples (right). **(B)** Boxplots represent the absolute difference between the score obtained from a specific pathway activation tool (GSVA, ssGSEA, zscore and plage) using all patients from the METABRIC dataset, and the score obtained when METABRIC is restricted to either Grade 1 (left), Grade 1 (middle), or Grade 3 (left) samples.

Supp. Figure 2 - Paquet et al.

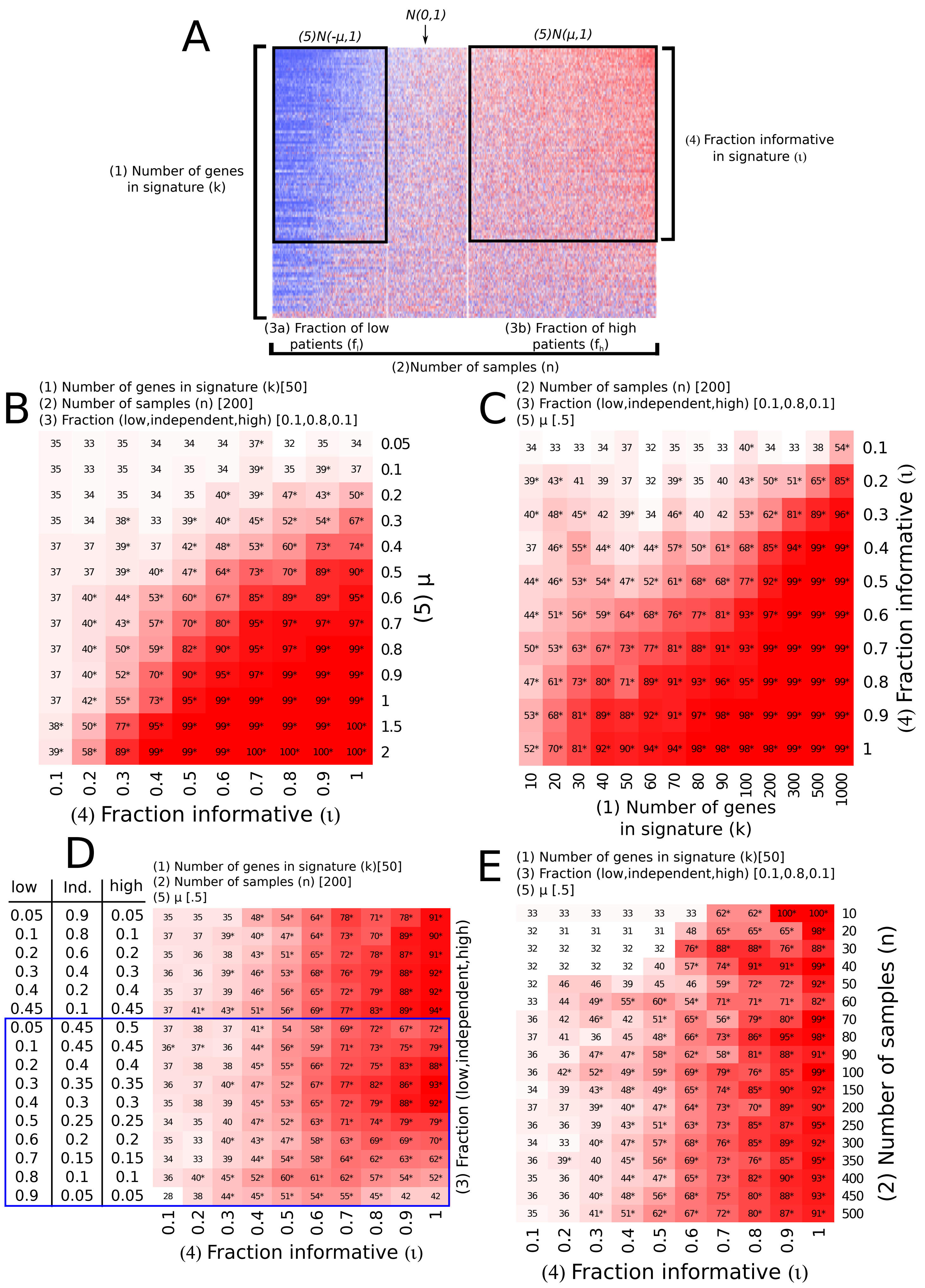

Supplementary Figure S2. The ROI$_q$ method is able to identify samples with either low or high activation (**A**) Description of the parameters utilized for the generation of the synthetic datasets. (**B-E**) Agreement (in percentages) between the ground truth and the estimated assignment obtained using the ROI$_{95}$ for different pairwise comparisons of parameters: (**B**) [4] Percentage of informative (ι) vs. μ [5], (**C**) [1] Size of signature (k) vs. [4] Percentage of informative (ι), (**D**) [4] Percentage of informative (ι) vs. [3] Size of ROI and (**E**) [4] Percentage of informative (ι) vs. [2] Number of samples (n). Stars denote parameter configurations that had statistically significant agreement between the ground truth and the estimation using the ROI$_{95}$ (Cohen's kappa statistics $P < 0.05$).

Supp. Fig. 3 - Paquet et al.

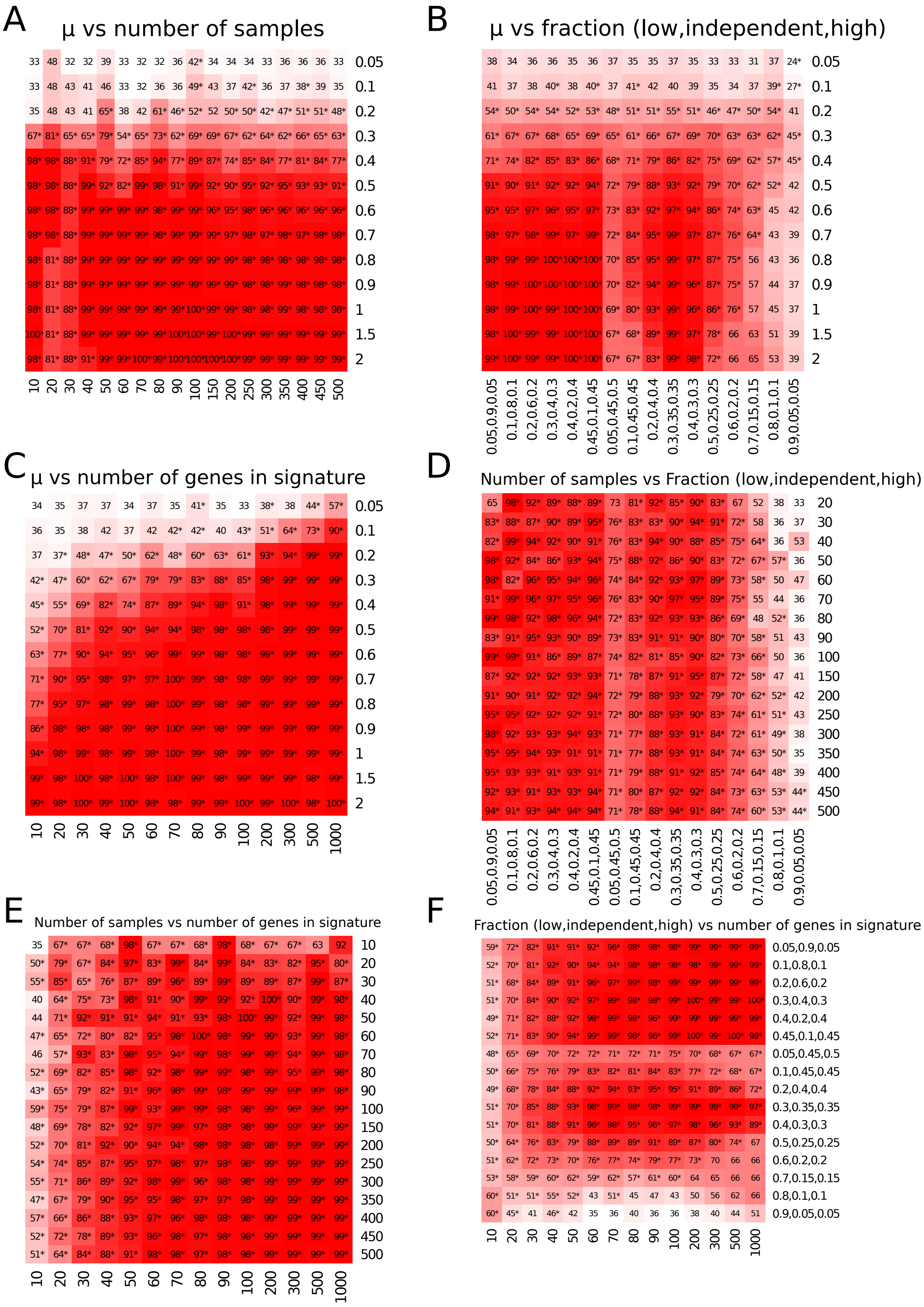

Supplementary Figure S3. The ROI_q method is able to identify samples with either low or high activation (**A-F**) Presentation of the agreement percentages between the imputed synthetic assignments and the one obtained using the ROI for different pairwise comparisons of two parameters: (**A**) μ vs. number of samples(n). (**B**) μ vs. size of the ROI. (**C**) μ vs. size of the signature (k). (**D**) Number of samples (n) vs. the size of the ROI. (**E**) Number of samples (n) vs. size of the signature(k). (**F**) Size of the ROI vs. size of the signature(k). Star following the agreement estimates highlight comparisons for which the agreement between the ROI and the real assignments are significant using a Cohen's kappa statistics $P < 0.05$.

Supp. Fig. 4. Paquet et al.

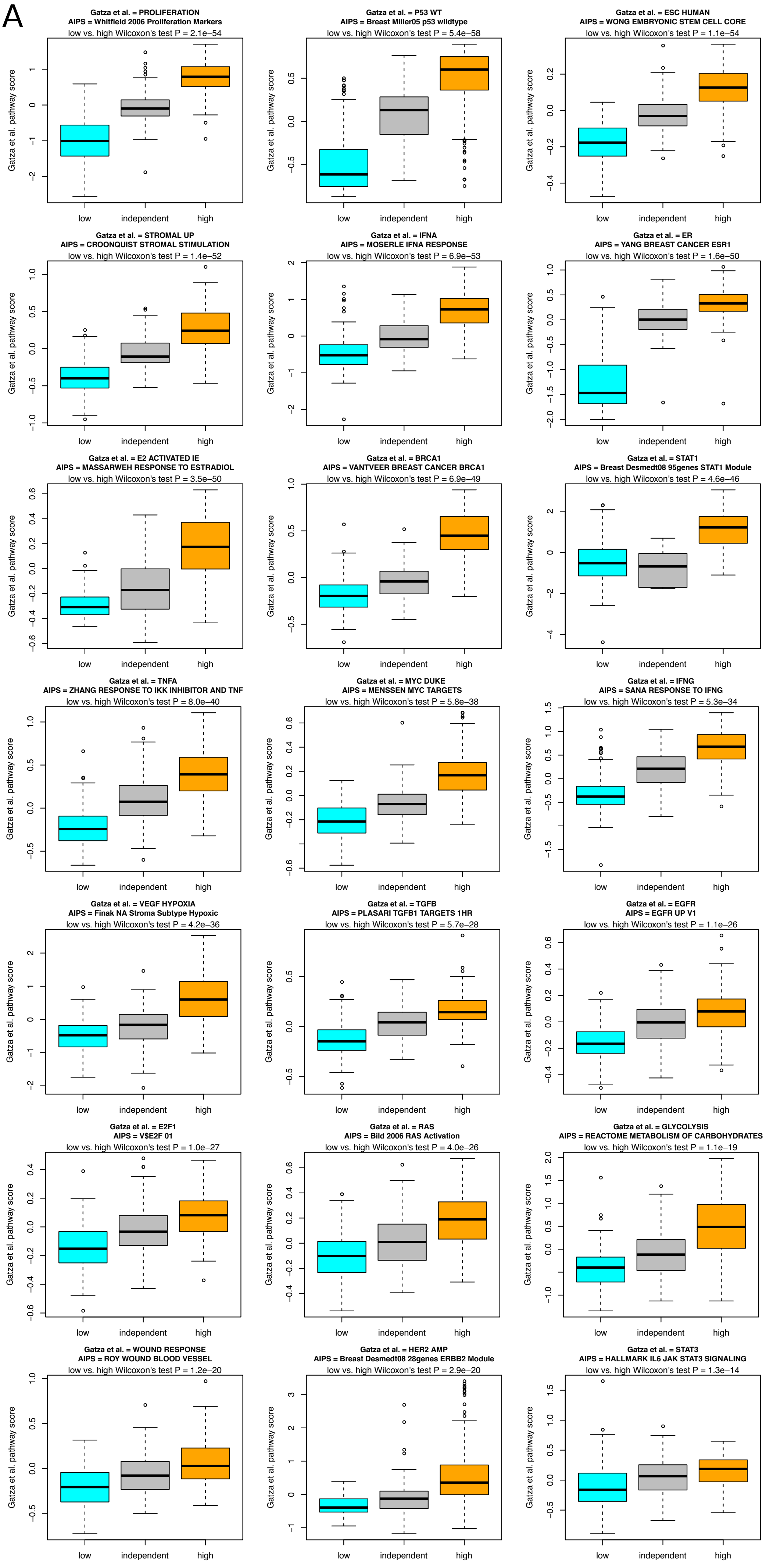

Supplementary Figure S4. Comparing pathway scores from Gatza et al. to AIPS assignments. (**A**) Boxplots displaying for every pathway the distribution of scores obtained for the AIPS low, independent and high assignments. The name of the Gatza et al. pathway is written on top of the boxplots as well as the corresponding AIPS signature and the result of a Wilcoxon's test comparing the scores distribution for the low and high AIPS assignments.

Supp. Fig. 5. Paquet et al.

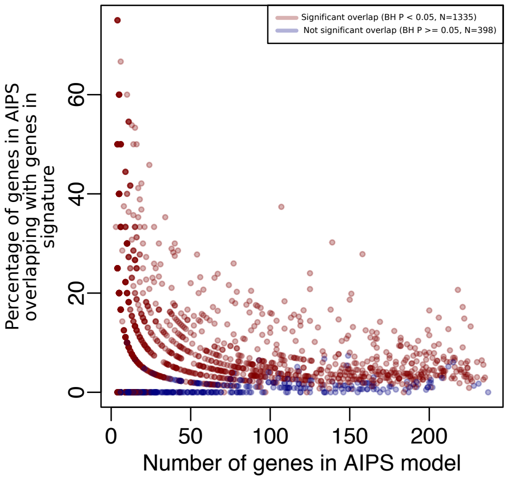

Supplementary Figure S5. Comparing genes selected in AIPS models with genes in the original gene signature. Plot showing the percentage of genes retained in AIPS models that overlap with the original signature in function of the number of genes in the AIPS models. The red dots represent the AIPS models for which we detect a significant overlap (Benjamini–Hochberg (BH) adjusted P < 0.05, 1335/1733 (77%)) and the blue dots are the AIPS models for which we detected no significant overlap (Benjamini–Hochberg (BH) adjusted P >= 0.05, 398/1733 (23%))
